# A window into living with an undiagnosed disease: illness narratives from the Undiagnosed Diseases Network

**DOI:** 10.1186/s13023-017-0623-3

**Published:** 2017-04-17

**Authors:** Rebecca C. Spillmann, Allyn McConkie-Rosell, Loren Pena, Yong-Hui Jiang, Undiagnosed Diseases Network, Christopher J. Adams, David R. Adams, Mercedes E. Alejandro, Patrick Allard, Euan A. Ashley, Mashid S. Azamian, Carlos A. Bacino, Ashok Balasubramanyam, Hayk Barseghyan, Alan H. Beggs, Hugo J. Bellen, Jonathan A. Bernstein, Anna Bican, David P. Bick, Camille L. Birch, Braden E. Boone, Bret L. Bostwick, Lauren C. Briere, Donna M. Brown, Matthew Brush, Elizabeth A. Burke, Lindsay C. Burrage, Katherine R. Chao, Shan Chen, Gary D. Clark, Joy D. Cogan, Cynthia M. Cooper, William J. Craigen, Mariska Davids, Jyoti G. Dayal, Esteban C. Dell’Angelica, Shweta U. Dhar, Katrina M. Dipple, Laurel A. Donnell-Fink, Naghmeh Dorrani, Daniel C. Dorset, David D. Draper, Annika M. Dries, David J. Eckstein, Lisa T. Emrick, Christine M. Eng, Cecilia Esteves, Tyra Estwick, Paul G. Fisher, Trevor S. Frisby, Kate Frost, William A. Gahl, Valerie Gartner, Rena A. Godfrey, Mitchell Goheen, Gretchen A. Golas, David B. Goldstein, Mary G. Gordon, Sarah E. Gould, Jean-Philippe F. Gourdine, Brett H. Graham, Catherine A. Groden, Andrea L. Gropman, Mary E. Hackbarth, Melissa Haendel, Rizwan Hamid, Neil A. Hanchard, Lori H. Handley, Isabel Hardee, Matthew R. Herzog, Ingrid A. Holm, Ellen M. Howerton, Howard J. Jacob, Mahim Jain, Yong-hui Jiang, Jean M. Johnston, Angela L. Jones, Alanna E. Koehler, David M. Koeller, Isaac S. Kohane, Jennefer N. Kohler, Donna M. Krasnewich, Elizabeth L. Krieg, Joel B. Krier, Jennifer E. Kyle, Seema R. Lalani, Lea Latham, Yvonne L. Latour, C. Christopher Lau, Jozef Lazar, Brendan H. Lee, Hane Lee, Paul R. Lee, Shawn E. Levy, Denise J. Levy, Richard A. Lewis, Adam P. Liebendorfer, Sharyn A. Lincoln, Carson R. Loomis, Joseph Loscalzo, Richard L. Maas, Ellen F. Macnamara, Calum A. MacRae, Valerie V. Maduro, May Christine V. Malicdan, Laura A. Mamounas, Teri A. Manolio, Thomas C. Markello, Paul Mazur, Alexandra J. McCarty, Allyn McConkie-Rosell, Alexa T. McCray, Thomas O. Metz, Matthew Might, Paolo M. Moretti, John J. Mulvihill, Jennifer L. Murphy, Donna M. Muzny, Michele E. Nehrebecky, Stan F. Nelson, J. Scott Newberry, John H. Newman, Sarah K. Nicholas, Donna Novacic, Jordan S. Orange, J. Carl Pallais, Christina G. S. Palmer, Jeanette C. Papp, Loren D. M. Pena, John A. Phillips, Jennifer E. Posey, John H. Postlethwait, Lorraine Potocki, Barbara N. Pusey, Rachel B. Ramoni Amy K. Robertson, Lance H. Rodan, Jill A. Rosenfeld, Sarah Sadozai, Susan L. Samson, Katherine E. Schaffer, Kelly Schoch, Molly C. Schroeder, Daryl A. Scott, Prashant Sharma, Vandana Shashi, Edwin K. Silverman, Janet S. Sinsheimer, Ariane G. Soldatos, Rebecca C. Spillmann, Kimberly Splinter, Joan M. Stoler, Nicholas Stong, Kimberly A. Strong, Jennifer A. Sullivan, David A. Sweetser, Sara P. Thomas, Cynthia J. Tifft, Nathanial J. Tolman, Camilo Toro, Alyssa A. Tran, Zaheer M. Valivullah, Eric Vilain, Tiphanie P. Vogel, Daryl M. Waggott, Colleen E. Wahl, Nicole M. Walley, Chris A. Walsh, Michael F. Wangler, Mike Warburton, Patricia A. Ward, Katrina M. Waters, Bobbie-Jo M. Webb-Robertson, Alec A. Weech, Monte Westerfield, Matthew T. Wheeler, Anastasia L. Wise, Lynne A. Wolfe, Elizabeth A. Worthey, Shinya Yamamoto, Yaping Yang, Guoyun Yu, Jing Zhang, Patricia A. Zornio, Kelly Schoch, Nicole Walley, Camilla Sanders, Jennifer Sullivan, Stephen R. Hooper, Vandana Shashi

**Affiliations:** 1 Division of Medical Genetics, Department of Pediatrics, Duke University Medical Center, Durham, NC USA; 2 Undiagnosed Diseases Network, NIH Common Fund, Bethesda, MD USA; 3 Department of Allied Health Sciences, University of North Carolina at Chapel Hill, Chapel Hill, North Carolina USA

**Keywords:** Undiagnosed, Patient narratives, Narrative typology, Undiagnosed diseases network

## Abstract

**Background:**

Patients’ stories of their illnesses help bridge the divide between patients and providers, facilitating more humane medical care. Illness narratives have been classified into three types: restitution (expectation of recovery), chaos (suffering and loss), and quest (unexpected positive effect from illness). Undiagnosed patients have unique illness experiences and obtaining their narratives would provide insights into the medical and emotional impact of living with an undiagnosed illness. Adults and children with undiagnosed diseases apply to be evaluated by the Undiagnosed Diseases Network (UDN). Written illness narratives from 40 UDN applicants, including 20 adult probands who applied for themselves and 20 parents who applied for their children, were analyzed for: 1) narrative content and 2) narrative type.

**Results:**

*Narrative content:* could be grouped into three themes: 1) Expectations of the UDN: the majority felt they had no further healthcare options and hoped the UDN would provide them with a diagnosis, with the adults expecting to return to their previously healthy life and the parents wanting information to manage their child’s healthcare. 2) Personal medical information: the narratives reported worsening of symptoms and some offered opinions regarding the cause of their illness. The proband narratives had few objective findings, while parental narratives had detailed objective information. 3) Experiences related to living with their undiagnosed illness: frustration at being undiagnosed was expressed. The adults felt they had to provide validation of their symptoms to providers, given the lack of objective findings. The parents worried that something relevant to their child’s management was being overlooked.

*Narrative type:* All the narratives were of the chaos type, but for different reasons, with the probands describing loss and suffering and the parents expressing fear for their child’s future. The parental narratives also had elements of restitution and quest, with acceptance of “a new normal”, and an emphasis on the positive aspects of their child’s illness which was absent from the probands.

**Conclusions:**

These narratives illustrate the chaos that coexists with being undiagnosed. The differences between the proband and parental narratives suggest that these two groups have different needs that need to be considered during their evaluation and management.

## Background

Undiagnosed diseases are common, affecting approximately 30 million Americans, and are associated with high rates of morbidity and mortality [1–3]. They include rare disorders which are difficult to recognize, atypical manifestations of more common disorders, yet to be described conditions and manifestations that cannot be explained medically. The majority (~80%) of undiagnosed diseases are believed to have a genetic etiology [4]. Undiagnosed disorders are often not amenable to the traditional diagnostic approaches and the lack of a diagnosis leads to repeated clinical consultations and laboratory testing, causing substantial personal and familial emotional and financial stress [1, 2, 5–11].

Both children and adults are affected with undiagnosed diseases, though possibly with different experiences. Parents of pediatric patients continue to struggle and make their way through the “diagnostic odyssey” of repeated clinical consultations and testing, with the purpose of finding information about their child’s long term health and recurrence risk information for the family [12]. In contrast, adults who have medically unexplained symptoms often feel that they need to continually legitimize their illness to medical providers and may find themselves in a situation where further diagnostic evaluation may not be offered to them [13, 14]. Thus, regardless of age, undiagnosed patients have extensive medical and psychological needs related to obtaining a diagnosis and appropriate management. Healthcare providers caring for undiagnosed patients also face challenges; needing extra time to parse out the symptoms and signs, determine if further diagnostic tests/procedures are necessary, initiate or participate in interdisciplinary provider communication and following up on results, all of which can be difficult to accomplish, even within a tertiary healthcare system. These multiple and unique challenges are elements that the Undiagnosed Diseases Network (UDN), funded by the National Institutes of Health, is designed to help manage. The UDN is a nationwide network-based research study established in 2014 whose purpose is to bring together clinical and research experts from across the United States to solve the most challenging medical mysteries using advanced technologies such as genome sequencing (https://undiagnosed.hms.harvard.edu). Currently, seven academic medical centers have been named as UDN clinical sites and receive applications for evaluation of undiagnosed adult and pediatric patients from all over the country. The review of each application prior to acceptance includes a comprehensive examination of the medical records for the medical history, family history and prior laboratory tests and procedures. Many UDN applicants whose medical records are under review contact the clinical sites to provide incremental bits of information through multiple emails and phone calls that they believe will facilitate their acceptance into the network. At the Duke University UDN clinical site a process was initiated to systematically request written narratives from all UDN applicants, to gather the patient/parent’s perspective of the illness and prior experiences all at one time.

Patient narratives, oral and written, are increasingly being recognized as important tools in modern medicine, providing the patient an opportunity to place their illness experience within the context of their life; additionally the narratives in and of themselves may have therapeutic value [15, 16]. For medical providers the narratives provide the opportunity to grasp and honor the meaning of the patient stories and act accordingly on their patient’s behalf in an engaged manner [17, 18]. Ground-breaking analyses of patients’ illness narratives by Frank [19] resulted in the identification of three types of narratives that offer insights into how patients experience illness and provide constructive methods for quality improvement in healthcare. The definitions of these narrative types have since been expanded, based on their application to different chronic illnesses, such as chronic fatigue syndrome, childhood cancer and medically unexplained symptoms [13, 20–22]. *The restitution narrative* is the least frequent among chronically ill individuals. This narrative reflects a transient nature of the illness with the patient expecting to recover from it. The narrative type usually occurs in the early stages of a chronic illness, before its longstanding nature becomes evident [21]. Restitution can also include the acceptance of a “new normal”, which may include acceptance of a negative outcome or an unresolved chronic health concern [22]. *The chaos narrative* is characterized by the concepts that life will never get better and no one is in control. This narrative reflects suffering, loss, inability to make plans, uncertainty, fear, rejection by clinicians and by others, is disjointed and disorganized, and can be difficult to hear and/or read [19]. In the literature it is exemplified by the narratives of adults with unexplained medical symptoms and chronic fatigue syndrome [21]. *The quest narrative* is one in which the patient sees the illness/disorder as a challenge and an impetus for change and believes that something can be gained through the experience. Additionally, parental quest narratives emphasize the positive attributes of their child and their gaining new parental and advocacy skills as a result of the child’s illness [22].

While these typologies provide a framework for the classification of illness narratives, they are often fluid with elements of more than one type found in the same story [19, 22]. The narratives may also transition from one type to another as the illness progresses or improves. Whitehead et al. in their analyses of interviews with 17 individuals diagnosed with chronic fatigue syndrome found that the narratives often started with restitution (individuals experienced symptoms with an assumption that with treatment, they would be healthy again) and then the narrative moved into chaos when symptoms got worse or did not resolve. Subsequently, the majority of the chaos narratives transitioned to the quest type, with the patients developing a positive outlook on their situation. Similarly, Bally et al. in their analyses of 16 parental narratives related to childhood cancer, found that these stories contained elements of all three types of narratives. The quest narratives of the parents included the positive effects of the child’s illness on their parenting and their gaining deeper appreciation for the child and family. Nettleton [13] [14] in their study of 18 adults with medically unexplained symptoms reported that living with an undiagnosed condition resulted in chaos due to ‘living with uncertainty;’ ‘issues of legitimacy;’ and a ‘resistance to psychological explanations of pain and suffering.’ Thus, illness narratives differ based on the type of illness (diagnosed or undiagnosed) and whether the narrative is being told by the affected individual or by a parent on behalf of their child.

Despite the increasing importance of illness narratives, no study has analyzed illness narratives of both adults and children who have an undiagnosed disorder/illness. To address this void, we conducted a retrospective study of written narratives of undiagnosed patients who applied to the UDN. Our objectives were: 1) To analyze the content of the narratives in the setting of whether the writer was an adult proband writing for himself/herself or a parent on behalf of their child, and 2) To analyze the structure of these narratives in the context of Frank’s narrative typologies [19]. Obtaining insights into the illness experiences of the UDN applicants would facilitate an individualized approach to these patients as well as inform the patient/provider communities of rare and undiagnosed diseases about the illness experiences of these individuals.

## Methods

### Patient selection

Each UDN applicant at the time they are assigned to the Duke clinical site is sent an email and is asked to “Please provide us with a one-page narrative telling us your story from your perspective. We have received the letter from your referring physician but we feel that we always learn so much when we hear the medical history directly from the patient.” The applicants are not provided with guidelines or specific topics to cover. An open-ended format was chosen, rather than a structured questionairre or interview, as it allows for flexibility and provides an opportunity for individuals to express their own views about their or their child’s illness [23, 24]. The completed narratives are sent to the team by email or fax. In order to achieve equal representation of the proband and parent narratives and to reduce bias, a purposeful sampling method was used to select the first 20 narratives written in each group (adult and parent) in the order in which they were received by the UDN research team. No distinction was made based on whether an application was accepted or rejected or if a decision was pending. Prior to analysis, each narrative was assigned a unique number, which was not linked to any identifiable information within the UDN or the order in which narratives were submitted.

### Narrative analyses

#### Narrative content (objective 1)

The narratives were analyzed using conventional content analysis with Atlas Ti (version 7; http://atlasti.com/). This qualitative approach allows for coding and subsequent themes to be developed directly from the data without the guidance of a theoretical model [25, 26]. All 40 narratives were first repeatedly read and discussed and then coded by AMR (has no contact with applicants and does not review medical records) and RCS (study genetic counselor). The coded data were then analyzed, emerging themes noted and the coded data sorted in support of each of the themes. This process was repeated and new codes developed as needed. Several steps were taken to ensure the validity and reliability of the coding and emerging themes. After initial coding was complete, codes were reviewed to determine agreement and discussed to resolve discrepancies each by AMR and RCS. The narratives were then independently read and summarized by a third investigator (VS). These summaries were then systematically reviewed by AMR to confirm consistency.

#### Narrative type (objective 2)

In order to categorize the narratives into the types proposed by Frank [19], we integrated the elements noted by Bally [22], Whitehead [21] and Nettleton [13]. We used this approach as the definitions presented by Frank were not developed for patients with an undiagnosed illness and were also based on the perspective of only the affected individual, without considering that an illness narrative may be written by a parent. Codes were developed incorporating the specific elements of the definitions.

#### Restitution

Restitution narratives include the concepts that the illness/disorder was initially perceived as transitory (i.e., previously healthy, now sick, with an expectation of health being restored) with the acceptance of a “new normal”, including acceptance of an ongoing illness/disorder.

#### Chaos

Chaos narratives lack organization in descriptions of illness and symptoms, searching for legitimacy of illness, uncertainty, fear, suffering, inability to consider a future or to make plans, and loss of control, self, or purpose.

#### Quest

Quest narratives include elements of a positive change because of the illness/disorder experience, the individual/parent taking control and advocating for themselves/their child, identification of new strengths, focusing on the positives attributes of self/child and hoping that through their illness experience something will be gained for others.

The codes that were developed from the previously described definitions were then used to categorize the narrative. The supportive codes and classifications of codes were then jointly reviewed by AMR and RSC. Each narrative was counted only once as having an element of a typology, regardless of how many times a code was present. Based on this process the different elements of a typology could be identified and a narrative could have elements of one or more types. Once this process was completed, the narratives were grouped based on whether it was written by the proband or a parent on behalf of the proband and examined for commonalities and differences. At the completion of the coding of these 40 narratives, as no new themes were emerging, we felt that saturation had been achieved. Quotes, illustrative of the themes and supportive codes were then extracted from the narratives and identifiers removed.

## Results

### Demographics

The Duke University UDN clinical site received narratives on 66 of the 81 applications received from November 2015 to August 2016. Forty consecutive narratives were chosen for analysis: of the 20 proband narratives one was from a minor proband who wrote her own narrative and of the 20 parental narratives, one was from a parent of an adult with intellectual disabilities who wrote on behalf of her child and one was from a parent when the adult child did not provide a narrative. We did not ask the parents who wrote the narrative for their child to specify their own gender, but it was evident in 12 cases that the narrator was the mother, 1 in which it was clearly the father; in the remaining 7, the gender of the parent could not be discerned. Additional demographics are in Table 1.

**Table 1:** Demographics of the Probands and Parents who wrote the narratives

	Proband (*n* = 20)	Parent (*n* = 20)
Gender of Proband		
Female	12	8
Male	8	12
Mean Age of Proband		
	42.15 ± 10.37 years	5.79 ± 4.68 years
Self-Reported Race/Ethnicity		
Caucasian	18	16
Black	1	1
Mixed Race	1	2
Non-Hispanic	19	19
Hispanic	0	1
Application Status		
Accepted	1	16
Not Accepted/Reconsidered	17	2
Decision Pending	2	2
Length of Narrative		
Longer than 1 page	10	13

The majority of the pediatric applicants (*n* = 13/20) had intellectual disability and one had experienced regression. All but one of the adults was cognitively normal.

### Narratives content

We identified three major themes that were common to the proband and parental narratives: We outline the commonalities in both followed by the differences between the proband and parent interviews for each of the three themes.

1. *Expectations of the UDN and importance of a diagnosis:*
  Commonalities: Both the proband and parent narratives expressed hope that the comprehensive nature of the UDN evaluations would lead to a diagnosis (17/40).
*…after undergoing genetic testing over the past year we are still without a diagnosis. It has become a situation of waiting to see what symptom manifests next to see if it yields any additional clues. I would be honored and eternally grateful to be included in what I believe is the best opportunity to finally know what exactly this is that has changed my life and my family’s life forever. (Narrative 21) Proband*
  The majority (*n* = 31/40) expressed that they had exhausted all other diagnostic options.
*I hope that I am accepted into this program since I just don’t know where to turn any longer. (Narrative 16) Proband*
  Distinctive features of Proband Narratives: In the proband narratives, for the majority the search for a diagnosis was a search for a cure, so that they could return to being well and to live the life they had prior to the onset of the illness.
*I want to make every effort I can to try to figure out what this illness is so I may work on getting well! (Narrative 15) Proband*
  Distinctive Features of Parent Narratives: Many of the parents (11/20) expressed that the lack of a diagnosis resulted in an uncertain future and wanted information that could improve the management and quality of life for their children:
*We don’t even expect a cure at this point. We just want to have some kind of an idea of what the future holds and to put a name to what is happening to our sweet boy. (Narrative 33) Parent*
2. *Personal Medical Information:*
  Commonalities: Much of the information contained in the narratives included the traditional components of an intake history such as the onset of symptoms, chief concern and symptom description, past medical history, family history, previous testing and procedures, treatments tried, and summaries of medical specialists’ evaluations. Some of the narratives (14/40) provided additional information that was not in the medical records and/or offered their own thoughts on possible causes of the illness.
*Another thing I’d like to point out as I am sure it is not in his file is the matter of his frequent low grade fevers. My suspicion for so long has been micro aspirations of reflux and vomit as the cause. (Narrative 33) Parent*
  Frequently (19/40) the narratives noted that symptoms were increasing in severity.
*I am worried this is getting worse, and I am not recovering. (Narrative 8) Proband*
*Since [my daughter] has started to have seizures bi-weekly to weekly basis. Lately, I see where she is having more seizures during the night time……. (Narrative 24) Parent*
  They described how medical providers had been unable to identify a unifying diagnosis or a medical explanation for their own symptoms or their child’s symptoms.
*No one knows why I have these symptoms. …. My PCP [primary care physician] is sending me to everyone she can think of to help me. (Narrative 10) Proband*
  Distinctive Features of Proband Narratives: The majority of proband narratives described symptoms of pain (13/20) and/or (9/20) fatigue, often with limited objective findings (17/20).
*The skin burning had become quite unbearable. Wearing a shirt was painful, my pants, shoes, earrings and putting makeup on my eyelids was 6–7 pain scale on 0–10. (Narrative 10) Proband*
  There were also descriptions of many different symptoms that were difficult to connect to one another.
*I have had issues with my eyes, hips, bones, hands and a general feeling of malaise. (Narrative 13) Proband*
  These narratives often describe a very specific event, which was seen as the triggering event or a moment when symptoms were noted when previously there were none.
My last healthy day was *month/*day/*year. (Narrative 10) Proband
  Distinctive Features of Parent Narratives: The parental narratives described more objective findings such as seizures, intellectual disability, and congenital heart disease. It was striking that many of the parent narratives were written with a high level of medical literacy, suggesting that parents were becoming “medicalized” through their experiences with their child’s illness/disorder.
*We started [my daughter] on [medication] and on day 2 of the medicine she had the respiratory side effect of bronchial swelling. If she would have had the 3* ^*rd*^ *dose I know she would not be here right now. Heart rate of 190–200 resting and 45–50 BPM [beats per minute] and retracting along with tracheal pulling and stridor. ….0*_*2*_ *is a PRN [pro re nata] around here. 95% of the time (she) is on room air and SATS [oxygen saturation] 98/110 resting. (Narrative 38) Parent*
  The parent narratives typically followed a chronological order, beginning with pregnancy and birth history, frequently describing their first concern about their child and how that occurred.
*We welcomed (our son) into the world after a normal pregnancy… He began to meet some of his first few milestones over the first few months. By month 5–6, we began to notice that [son] was falling behind in his milestones – he was not progressing with crawling, he could not roll over with consistency, could not track things visually, seemed to have difficulty with recognizing sounds, and did not have much verbal communication. (Narrative 36) Parent*
3. *Experiences related to living with their undiagnosed illness:*
  Commonalities: The process and opportunity to “tell their own story” seemed to be appreciated by both the probands and parents.
*We appreciate your time in reading our concerns. (Narrative 26) Parent*
*Thank you for taking time to review my health history. This document contains my story from the last three and a half years. (Narrative 14) Proband*
  And for some, the process of revisiting the story was emotional.
*As I sit to summarize [daughter] I find myself tearing up and very emotional. Nine years of chronic medical crisis’ and so many failed treatments leaves so much to recap. (Narrative 38) Parent*
  Both proband and parent narratives described how emotionally challenging it was to continue to look for answers.
*The years of searching for answers that have only lead to more questions have been extremely challenging. (Narrative 21) Proband*
*Advocating for her to have the best quality of life and to help improve her medically has been an extremely challenging and tiring process. I have to set limits on what is realistic in this journey. It is like I get on a hamster wheel and can’t get off. (Narrative 38) Parent*
  Both proband and parent narratives described previous experiences with health providers and diagnostic evaluations, including frustration with the process and lack of a diagnosis.
*I saw twenty doctors who could not help me. I slowly went up and down with some recovery to feeling sick over the course of 14 years. (Narrative 9) Proband*
  Distinctive Features of Proband Narratives: The source of the frustration found in the proband narratives for many, was that multiple medical evaluations only led to disappointment since no definite findings had been identified to explain the symptoms and laboratory testing did not validate their symptoms. In a few (3/20) there was expressed validation at an abnormal finding that confirmed that their symptoms were real.
The last neurologist I saw ran a *** that had abnormal results…. He also ordered nerve conduction studies that I was told showed slower conduction. I felt redeemed.
*(Narrative 5) Proband*
  And some (*n* =3/20) probands reported either being told of the possibility of or the recommendation for investigation of a psychological component to their symptoms.
*My current PCP, had me trial (medication) thinking that stress could be a contributing factor. (Narrative 11) Proband*
  They also expressed that health providers don’t ‘hear” them or they felt misunderstood, or were labeled as “difficult”.
*…My neurologist has basically told me that I am a difficult case and there is nothing to point towards a neurologic disorder. (Narrative 4) Proband*
  Treatment attempts were reported as not successful or to result in only marginal improvements for a short time. Probands also expressed dissatisfaction if they felt they needed additional or specific tests to diagnose their disorder and the health provider disagreed.
My symptoms not only persisted but worsened. I was sent to ***. That neurologist also ran no tests. After a brief exam, he turned to his fellow and said, “Tell her she’ll live,” and strode out of the room. (Narrative 5) Proband
  The overwhelming majority of the proband narratives reported loss; loss of the person they used to be and being unable to work or enjoy life since the onset of their illness or disorder. Many provided specific examples of athletic ability, professional or career success that have been negatively affected and being unable to meet family responsibilities.
*Since my illness, I am unable to do a lot of the things I used to enjoy. Working a normal job has become virtually impossible. Enjoying social and physical activities has also been more difficult. (Narrative 13) Proband*
*Prior to this, I was healthy and active, ran marathons and worked long hours as a [professional]. (Narrative 7) Proband*
  Distinctive Features of Parental Narratives: The parents’ narratives differed from the proband narratives regarding the source of their frustrations. Although a few of the parent narratives also described not being “heard” by the health providers, the parents expressed worry about the possibility that something would be overlooked that might be important, and that physicians had reached the limit of what they could offer.
*So many physicians dismissed things because she “looked” okay when she was little or later because she was outside of their comfort level…. I commonly get the “she is beyond me” answer or that the physician relies on me as I know her so well… This is where the frustration always comes into play. (Narrative 38) Parent*
  The majority of parents (14/20) described the complexity of care required for daily living for their child*.*
*She has eight standing appointments each week (PT [physical therapy], OT [occupational therapy], Swallow therapy, homebound school, and craniosacral therapy). She also sees peds [pediatrician], neuro [neurology], gastro [gastroenterology], a dietician, ophthalmology, genetics and an orthopedic specialist regularly. (Narrative 37) Parent*
  For some of the parents, there was an expressed helplessness and distress at being unable to improve their child’s outcome.
*He was wasting away in front of us and nothing we did seemed like it could stop it. (Narrative 23) Parent*
*I can’t explain the fear and rush of emotions we felt as parents knowing nothing about seizures or Epilepsy at the time. We truly thought we were losing him. (Narrative 32) Parent*
  The majority of the parent narratives emphasized positive attributes of the child when describing how the illness/disorder has affected their child.
*My son has suffered but yet he doesn’t complain. He is the picture of a strong and kind young man. He has grace and courage where others would and do fail to. He deserves a chance at having answers or paving the way for answers for others. (Narrative 23) Parent*
  In these descriptions, the love for the child comes through.
*But our sweet boy, he’s just so precious. He is such a sweetheart. He gives the best hugs and snuggles. He is absolutely adorable when he smiles at you. He is easy-going and hard-working and laid-back and persistent. He is so resilient. He rarely gets frustrated. He is a pleasure to be around and brings joy to our lives. I just wish we could figure out what is going on. (Narrative 31) Parent*
  The parent narratives suggested that parents are advocates for their children and are determined to do everything they can to help improve their child’s quality of life, even if there is no cure.
*As parents we want nothing more in the world to figure out what is going on with her. … She has determination that is mind blowing, she is our hero. (Narrative 25) Parent*
  In a few of the parent narratives we also found descriptions of an unexpected positive change resulting from the illness/disability.
*She is truly a gift that has taught me the true meaning of unconditional love. (Narrative 38) Parent*

### Narrative typology

We found that all 40 narratives were of the chaos type (Table 2). However, the source of the chaos differed based on whether the narrative was written by a proband or a parent on behalf of their child. The majority of the proband narratives were exclusively of the chaos type (*n* = 19/20), while the majority of the parent narratives included additional characteristics of quest (*n* = 17/20) or restitution (*n* = 10/20). Ten of the parent and one of the proband narratives had elements of all three typologies. The majority of the proband narratives did not include the additional elements of quest and/or restitution. The narrative typologies closely mirrored the lived illness experience theme in the content of the narratives and thus we summarize the narrative types in the table, without including overlapping quotes.

**Table 2:** Narrative typologies and supporting codes

	Proband	Parent
Restitution Narrative (total)	4	10
Codes		
Healthy, sick, with expectation that health would be restored	3	3
Acceptance of a new normal inclusive of the illness/disorder	1	7
Codes		
Chaos Narrative (total)	20	20
Disorganization in description of illness and/or symptoms	14	2
Suffering and loss	19	9
Fear and uncertainty/difficulty making future plans	9	16
Searching for legitimacy	7	1
Codes		
Quest Narrative (total)	1	17
Focus on the positive attributes of self/child/family		16
Individual/parent taking control and advocating/identification of new strengths		14
Hope that something will be gained for others	1	

## Discussion

This study analyzed for the first time, the illness narratives pertinent to both adults and children with undiagnosed diseases. Overall, the burden of living with an undiagnosed condition is high, with suffering, frustration and uncertainty. We found both commonalities and important differences in the content of the narratives and in the typologies between the adult proband and parental narratives.

### Narrative content

Not surprisingly, both the probands and the parents applied to the UDN after having exhausted all other options. While both groups had high expectations of the UDN, the probands wished to resume their former lives, while the parents recognized that their child was unlikely to completely recover and wanted information how to better medically manage their child. This may be a reflection of the early onset and severe manifestations seen in children with difficult to diagnose disorders; for the probands who often developed symptoms abruptly, a clear awareness of the demarcation between being well and falling sick persisted, such that they yearned to become well again.

The medical information in both groups reflected important reasons for why they applied to the UDN- for many the symptoms were getting worse and for all there was no answer. A striking difference between the proband and parental narratives was the lack of objective findings in most of the proband narratives, in contrast to the detailed objective findings in the parental accounts, again reflective of the multiple and serious features of pediatric undiagnosed diseases. The narratives were especially informative in providing information that was not in the medical records, such as the proband/parent’s impression of potential causes/diagnoses or a more accurate timeline of when certain events or symptoms presented. This information could be used by the UDN team to either obtain additional records that could be relevant or to address these problems specifically during the review and evaluation process.

The narratives provided insight into the lived illness experiences of the proband or the experience as described by the parent. Narrators appreciated being given an opportunity to tell their story in their own words, perhaps attesting to the power of narrative medicine [17]. Parental narratives are widely accepted as giving voice to the child’s experience, which may go unsaid otherwise and so are as valuable as the proband narratives [16]. A commonality in both the proband and parent narratives was the feeling of not being heard by their medical team and/or being categorized as a difficult patient or parent. While the term “difficult patient” has a negative connotation, a patient may be perceived as difficult because the health care provider cannot identify the problem, is unable to diagnose the disorder, and/or is unable to help the patient to improve their health outcome [27]. These are characteristics of the undiagnosed patient which may contribute to their perception of not being heard. Health care providers and in particular, genetic counselors, are positioned to help facilitate medical care for these patients and should consider the emotional component associated with being undiagnosed, thus meeting their needs with understanding and empathy.

### Narrative typologies

Analyses of the 40 narratives revealed that all were of the chaos type, but elements of all three of Frank’s types of illness narratives (restitution, chaos, and quest) were present in a fourth of the narratives—more so in the parental narratives, with only a few of the proband narratives reflecting restitution and only one quest. However, the restition in the proband narratives was exclusivly related to the initial expectation that health would be restored. The narratives then became dominated by chaos, when health was not restored. The chaos in the proband narratives was mainly due to the suffering and loss (illustrations of life before the onset of illness and what had been lost: athleticism, employment and professional successes) and disorganization of the narrative with a multitude of difficult to connect symptoms. This disorganization is consistent with Frank’s description of chaos being “beyond words” [19]. It is also possible that because they are undiagnosed, that the probands are concerned about “leaving something out” that may be the key to their returning to their previous lives resulting in their providing massive amounts of unrelated details and so chaos overtakes the narrative. Our findings suggest that living with an undiagnosed condition prevents the probands from being able to transition out of chaos to quest and the very nature of chaos prevents them from being able to clearly communicate their illness story.

In contrast, the chaos associated with the parent narratives was related to fear and uncertainty about their child’s future and an inability to make plans. Most of the research on parental narratives has focused on diagnosed childhood chronic illness or cancer and thus these experiences are different from those of parents of children who apply to the UDN [22, 28]. For childhood cancer for example, although there is uncertainty about the future, there is a definitive diagnosis, a network of other parents who have children with the same diagnosis, and a plethora of support, resources, and possible next steps that are outlined by their healthcare providers. Bally describes that the chaos for parents of children with cancer occurred typically during the treatment phase with the transition to quest and restitution occurring after treatment [22]. It is interesting that in our study, the parental narratives were found to be chaotic, but still had elements of both quest (finding new parenting strengths, focusing on the positive) and restitution (acceptance of a new normal), despite the lack of a diagnosis. The restitution and quest found in the parent narratives tempered the chaos associated with the fear and uncertainty that comes with having a sick child.

### Clinical implications

The UDN clinical teams are put in the difficult position of having to make a decision regarding acceptance of applications from patients/parents who believe the network is their last hope for help. The decision is made almost exclusively by medical record review with very little, if any, interaction with the applicant prior to a decision. In general, if an applicant is not accepted to the UDN (due to different reasons, including the lack of objective findings), there could be very little closure for the applicant and the belief of not being heard may be reinforced. While indicating that acceptance was not appropriate, an acknowledgement of the pain and suffering that had taken place would be important. This may be helpful to some of the adult probands in their transition from chaos to quest or restitution- acceptance of a new normal. For parents of children with undiagnosed illness, reassurance that they have done everything they possibly could have for their child’s medical care and encouraging their role as an advocate for their child are important. It may also be important to consider inclusion of a social worker or another mental health provider to facilitate communication with the applicants.

### Limitations of the study

This was a retrospective analysis of self- reported and open-ended patient/parent narratives and since it was not an interview, in which follow-up questions could be asked, there may be aspects of the illness story that could be missing. As these narratives were in a written format, the proband or parent would have had the opportunity revise or review their narrative prior to it being submitted and thus relevant information could have been lost/edited in the revision process. Additionally, there may be differences in an individual’s ability to write as opposed to verbally tell a story.

## Conclusions

The written narratives of UDN applicants, both probands and parents, are powerful and allow for an initial connection with the medical team. Important medical information and a chronology of events may be gleaned from the narratives. It is evident that having an undiagnosed disease results in emotional distress, in addition to suffering from the symptoms and signs of the illness. Probands and parents differ in their experiences with the undiagnosed disease and their expectations of the UDN. Further study of the psychological aspects of undiagnosed diseases is slated to be an important future direction for the UDN [29] and this could lead to more individualized patient centered assessments.

## Abbreviations

BPM: Beats per minute
Gastro: Gastroenterology
Neuro: Neurology
OT: Occupational therapy
PCP: Primary care physician
Peds: Pediatrician
PRN: Pro re nata “as needed”
PT: Physical therapy
SATS: Oxygen saturation
UDN: Undiagnosed diseases network
